# Altered brain function associated with non-suicidal self-injury in adolescents with major depressive disorder: An rs-fMRI study

**DOI:** 10.1371/journal.pone.0356601

**Published:** 2026-08-20

**Authors:** Hongjuan Bai, Shaohua Chang, Jing Li, Ning Li, Hailong Wang, Xinyu Du, Hongyan Zhao, Xuebing Xu

**Affiliations:** 1 Ningxia Ningan Hospital (Ningxia Provincial Mental Health Center), Yinchuan, Ningxia, China; 2 Department of Magnetic Resonance Imaging, Ningxia Ningan Hospital (Ningxia Provincial Mental Health Center), Yinchuan, Ningxia, China; 3 Ningxia University, Yinchuan, Ningxia, China; Universita Cattolica del Sacro Cuore Sede di Roma, ITALY

## Abstract

**Background:**

Nonsuicidal self-injury (NSSI) is prevalent and harmful among adolescents with major depressive disorder (MDD), threatening mental and physical health. This study aims to elucidate the poorly understood neural mechanisms underlying NSSI behaviors and motivations in this group.

**Methods:**

133 adolescent participants were recruited, comprising 45 MDD with NSSI, 46 MDD without NSSI, and 42 age- and sex-matched healthy controls. All underwent resting-state functional MRI, from which low-frequency amplitude fluctuations (ALFF) were assessed and regions showing group differences were used as seeds for functional connectivity (FC) analyses. Associations between ALFF/FC values and NSSI behaviors and motivations were then evaluated via a cross-validation prediction method for robustness and generalizability.

**Results:**

Adolescents with MDD who engaged in NSSI showed increased ALFF in the left precuneus, significantly associated with NSSI behaviors, and increased left precuneus–left cuneus FC, significantly associated with behaviors and intrapersonal negative reinforcement motivations. Prediction analyses confirmed the robustness of these associations, with ALFF (*r*_(predicted, observed)_ = 0.653, *p* = 0.001) and FC (*r*_(predicted, observed)_ = 0.601, *p* = 0.001; *r*_(predicted, observed)_ = 0.587, *p* = 0.001).

**Conclusion:**

Our study identified key alterations in brain activity associated with NSSI behaviors and motivations in adolescents with MDD, providing neural markers for NSSI prediction and diagnosis and targets for precision interventions.

## 1. Introduction

Non-suicidal self-injury (NSSI) refers to the deliberate infliction of direct damage to one’s body tissue, such as cutting, hitting, or burning, in the absence of suicidal intent, typically defined as occurring on at least five occasions within the past year [1]. With a prevalence of approximately 18% [2], NSSI is widespread in community, school, and clinical populations, with notably higher rates among individuals with psychiatric disorders and adolescents. It has emerged as a pressing global public health concern.

Among psychiatric populations, NSSI is particularly prevalent in individuals with major depressive disorder (MDD) [3–6]. MDD is characterized by marked and persistent low mood, psychomotor retardation, and diminished activity [7]. Patients with MDD are nearly twice as likely to engage in NSSI as those without psychiatric disorders [8]. However, despite the growing body of literature on NSSI, much of the evidence has been derived from non-clinical samples, limiting its applicability to clinical populations [9]. Clinically, the comorbidity of MDD and NSSI not only increases disease burden but also exacerbates emotional dysregulation and elevates suicide risk.

Adolescence is a critical developmental period marked by rapid growth in self-awareness and immature emotional regulation, making individuals more vulnerable to NSSI. Across the lifespan, NSSI prevalence peaks during adolescence, especially in those with MDD [5]. In countries such as China and Canada, the prevalence of adolescent NSSI is approximately 30% in non-clinical samples but rises to 62–77% in clinical samples with depression [10,11]. This pattern is driven by the strong association between depressive symptoms and NSSI [12], which form a mutually reinforcing cycle that exacerbates psychiatric symptoms and self-injurious behaviors [13–15]. NSSI significantly compromises adolescents’ physical and mental health as well as social functioning, exerts long-term negative effects persisting into early adulthood [16,17], and markedly increases the risk of subsequent suicide [18,19]. Adolescence is also a period of heightened neuroplasticity, which supports adaptive changes but may also increase vulnerability to mental health problems [20]. Neurodevelopmental changes during this stage can disrupt the maturation of emotional and behavioral regulation, potentially forming a biological basis for NSSI [21]. Nevertheless, the relatively small body of empirical research on the neurobiological mechanisms of NSSI means that our understanding of this phenomenon remains limited.

In recent years, resting-state functional magnetic resonance imaging (rs-fMRI) has been increasingly employed to investigate the neural mechanisms underlying suicide and self-injury. Studies have reported abnormal brain activity or connectivity in the prefrontal cortex, limbic system, right caudate nucleus, and precuneus in patients with MDD who engage in NSSI [22–24]. Moreover, the default mode network (DMN) has been closely linked to suicide and self-injury. The DMN—comprising the medial prefrontal cortex, posterior cingulate cortex (PCC), precuneus, and posterior medial parietal cortex—is more active at rest than during task engagement [25] and plays a pivotal role in self-referential processing, emotion regulation, and introspection. As core nodes of the DMN, the PCC and Precuneus have shown reduced functional connectivity in adolescents with suicidal tendencies [26]. Zhou et al. further identified DMN and visual network (VN) related brain alterations associated with NSSI in adolescents with MDD [27]. However, some studies have reported findings that contradict these results [28], which may largely be attributed to differences in study populations, NSSI scales, and neuroimaging indices. Overall, neuroimaging research on the neural mechanisms of NSSI in adolescents with MDD remains scarce, and current findings are inconsistent. Notably, most existing studies have focused on the behavioral characteristics of NSSI—such as frequency and methods—while paying little attention to its motivations. NSSI typically serves multiple motivations, including intrapersonal motivations such as emotion regulation and self-punishment, as well as interpersonal motivations such as expressing distress, influencing others, or punishing others; intrapersonal motivations account for approximately 66%–81% of cases [29]. Examining NSSI from a motivational perspective may help elucidate the relationship between its underlying motivations and neural mechanisms, thereby improving the precision and personalization of clinical interventions.

Moreover, traditional neuroimaging analyses typically rely on in-sample associations between imaging indices and behavioral or cognitive measures, which are often constrained by sample-specific characteristics and therefore have limited ability to generalize beyond the studied cohort. To enhance the robustness and external validity of the findings, we further employ a machine learning–based cross-validated approach to evaluate the stability and generalizability of brain–behavior associations. Specifically, based on previous methodological experience [30–32], we employed 4-fold cross-validation using linear regression models to test whether associations between brain measures and NSSI behaviors and motivations could be generalized to unseen individuals. In contrast, cross-validation explicitly evaluates model performance on independent data partitions, thereby providing an estimate of predictive validity for new participants’ behavior [33]. This approach has gained increasing recognition in cognitive neuroscience and is widely used to improve the reproducibility and robustness of brain–behavior association studies [31].

Building on this framework, the present study integrates resting-state functional MRI (rs-fMRI) techniques and related brain functional metrics, together with a machine learning–based cross-validation approach, to investigate the neural mechanisms underlying non-suicidal self-injury (NSSI) behaviors and their motivations in clinical adolescents with major depressive disorder (MDD). The aim is to provide a neuroscientific basis for the prediction and identification of NSSI, as well as to identify potential neural targets for precise interventions in this population.

## 2. Materials and methods

### 2.1. Participants

Participants with MDD were recruited from a public psychiatric hospital in western China, and age- and sex-matched healthy control (HC) participants were recruited from the local community between January 2024 and February 2025. All participants underwent diagnostic interviews conducted by psychiatrists who had received standardized, independent training, following the Diagnostic and Statistical Manual of Mental Disorders, Fifth Edition (DSM-5) [34] Standardized computerized psychometric assessments were administered with professional assistance. Inclusion criteria were as follows: (1) being right-handed adolescents (12–24 years), with the age range defined according to Sawyer et al. [35] and aligned with the academic developmental stages of Chinese adolescents; (2) for MDD participants, meeting DSM-5 criteria for depressive disorders, with no prior history of antidepressant use, transcranial magnetic stimulation, electroconvulsive therapy, or psychotherapy; (3) for MDD participants with NSSI, meeting DSM-5 criteria, operationalized as engaging in NSSI on ≥5 days in the past year and at least once in the past month, whereas participants without NSSI had no history of self-injury; (4) for HC participants, having no history of any mental health disorder and NSSI; (5) completing the diagnostic interview, psychometric assessments, and MRI scanning; (6) meeting MRI safety requirements (no history of traumatic brain injury, no cardiac pacemaker, artificial joints, or other metallic implants, and no claustrophobia). Written informed consent was obtained from all participants; for those under 18 years of age, consent was additionally obtained from a legal guardian.

Following these procedures, a total of 163 individuals were initially recruited. Sixteen were excluded due to incomplete assessments or fMRI scans, and fourteen were excluded because of poor fMRI data quality or excessive head motion. The final sample included 133 participants, consisting of 72 females and 61 males, with a mean age of 16.24 ± 2.69 (*M* ± *SD*). The participants were divided into three groups: MDD with NSSI (*n* = 45), MDD without NSSI (*n* = 46), and HC participants with neither MDD nor NSSI (*n* = 42). Demographic characteristics for each group are presented in Table 1.

**Table 1:** Demographic, clinical, and head-motion characteristics and group comparisons.

Characteristics	MDD with NSSI (*n* = 45)	MDD without NSSI (*n* = 46)	HC (*n* = 42)	*χ*²/F	*p*-value	η^²^_p_/Cramer’s V
Gender (female/male)	26/19	19/23	27/19	1.965	0.374	0.122
Age (years)	15.62 ± 2.38	16.19 ± 2.62	16.89 ± 2.95	2.595	0.079	0.038
SDS	62.22 ± 10.18	59.48 ± 11.89	30.28 ± 6.79	199.528	< 0.001	0.695
NSSI-B	23.51 ± 9.87	0	0	–	–	
NSSI-M						
SPR	18.20 ± 8.95	–	–	–	–	
INR	9.80 ± 4.28	–	–	–	–	
EE	8.16 ± 3.67	–	–	–	–	
Head Motion	0.12 ± 0.04	0.11 ± 0.04	0.12 ± 0.04	0.235	0.791	0.004

*Note*. SDS = Self-Rating Depression Scale score; NSSI-B = Adolescent Non-suicidal Self-injury Assessment Questionnaire behavior subscale score; NSSI-M = Adolescent Non-suicidal Self-injury Assessment Questionnaire motivation subscale score; SPR = social-positive reinforcement; INR = intrapersonal-negative reinforcement; EE = emotion expression; Head motion = mean framewise displacement (Jenkinson).

### 2.2. Clinical assessment

#### 2.2.1. The Zung Self-Rating Depression Scale (SDS).

Depression severity was assessed using the Zung Self-Rating Depression Scale (SDS) [36], which consists of 20 items (e.g., “I feel down-hearted and blue”). Participants rated each item on a 4-point Likert scale reflecting their feelings over the past week, with higher scores indicating greater severity of depressive symptoms. The SDS was introduced in China in 1984 [37] and has since been widely validated and frequently used in clinical assessments of depression [38–40]. In the present study, the scale demonstrated excellent internal consistency, with a Cronbach’s *α* of 0.941. Full item content and the Chinese version of the scale are provided in Supporting information S1 Table.

#### 2.2.2. The Adolescent Non-suicidal Self-injury Assessment Questionnaire (ANSAQ).

NSSI over the past year was assessed using the Adolescent Non-suicidal Self-injury Assessment Questionnaire (ANSAQ) [41]. The ANSAQ is a self-report measure specifically developed for Chinese adolescents, integrating DSM-5 recommendations for NSSI diagnosis with items from existing NSSI assessment scales. It consists of two subscales: behavior and motivation. The behavior subscale includes 12 common forms of adolescent NSSI, ranging from behaviors causing no visible tissue damage to those resulting in clear tissue injury, such as “deliberately pinching oneself” and “deliberately biting oneself.” Items are rated on a 5-point scale (0–4), where 0 indicates absence of the behavior and higher scores reflect greater severity of NSSI. Participants without any NSSI behavior scored 0 on all items of the behavior subscale. The motivation subscale comprises 19 items rated on the same 5-point scale and is divided into three dimensions: (1) social-positive reinforcement (SPR), reflecting NSSI performed to achieve a favorable state or fulfill social needs (e.g., “to attract others’ attention”); (2) intrapersonal-negative reinforcement (INR), reflecting NSSI aimed at alleviating or escaping from negative states (e.g., “self-punishment or atonement”); and (3) emotion expression (EE), reflecting NSSI performed to express personal emotions (e.g., “to express anger”). Full item content and the Chinese version of the scale are provided in S2 Table.

The ANSAQ has been widely used and validated [41–43]. In the present study, the behavior subscale demonstrated excellent internal consistency (Cronbach’s *α* = 0.926). The motivation subscale also showed good reliability (Cronbach’s *α* = 0.886), with the three dimensions exhibiting α coefficients of 0.810, 0.873, and 0.792, respectively.

### 2.3. Imaging data acquisition and preprocessing

#### 2.3.1. Data acquisition.

All participants underwent a 5-minute structural MRI scan followed by an 8-minute resting-state functional MRI (rs-fMRI) scan. Imaging data were acquired using a GE 1.5T scanner (SIGNA Explorer, GE Healthcare, USA). Prior to the formal scans, each participant completed a mock scanning session to familiarize themselves with the scanning environment and minimize head motion. During the scans, participants’ heads were comfortably positioned and stabilized using foam pads, and earplugs were provided to reduce noise. Participants were instructed to lie still with eyes closed, remain relaxed and awake, avoid deliberate thoughts, and not fall asleep. After scanning, participants were asked whether they had fallen asleep during the scan.

Resting-state functional images were acquired using a gradient-echo echo-planar imaging (GRE-EPI) sequence with the following parameters: repetition time (TR) = 2000 ms; echo time (TE) = 30 ms; flip angle (FA) = 90°; field of view (FOV) = 260 mm × 260 mm; matrix = 80 × 80; slice number = 33; slice thickness = 3.5 mm; distance between slice = 1 mm; voxel size = 3.5 mm × 3.5 mm × 3.5 mm. A total of 240 consecutive volumes were collected. Structural images were acquired using a 3D BRAVO (Brain Volume Imaging) sequence, a high-resolution T1-weighted imaging (T1-TWI) method optimized by GE, based on an inversion recovery fast spoiled gradient echo (IR-FSPGR) sequence. Compared with conventional T1-TWI, this sequence provides higher spatial resolution and more uniform gray-white matter contrast, improving brain tissue segmentation, registration accuracy, and enhancing the reliability of functional connectivity analyses. Structural scan parameters were: TR = 7.2 ms; TE = 2.8 ms; FA = 12°; FOV = 224 mm × 224 mm; matrix = 224 × 224; slice thickness = 1 mm; distance between slice = 1 mm; voxel size = 1 mm × 1 mm × 1 mm. All scans were performed by trained professionals.

#### 2.3.2. Data preprocessing.

All imaging data were preprocessed on Matlab R2022a [44] using the DPABI V5.4 toolbox [45,46], based on SPM12 [47]. The preprocessing steps included: (1) format conversion: raw data were converted to NIFTI format; (2) removal of initial time points: the first 10 volumes of each participant’s time series were discarded to stabilize early signals and account for scanner adaptation noise; (3) slice timing: resting-state data were acquired using an interleaved accelerated sequence, and for each participant, slice timing was determined in Matlab with the middle slice as the reference for temporal alignment; (4) head motion correction: According to the recommendation of Power et al. [48], participants with framewise displacement (FD) > 0.2 mm were excluded; (5) normalization: functional images were normalized to MNI space using DARTEL, with each participant’s structural image first coregistered to the mean functional image, then segmented into gray matter, white matter, and cerebrospinal fluid to generate flow fields, which were applied to normalize functional images to the MNI template with a resampled voxel size of 2 mm × 2 mm × 2 mm; (6) smoothing: images were smoothed using a Gaussian kernel with full width at half maximum (FWHM) of 6 mm × 6 mm × 6 mm; (7) detrending: linear trends accumulated during scanning were removed; (8) regression: signals from white matter, cerebrospinal fluid, and 24 head-motion parameters (six motion parameters at the current time point, six from the previous time point, and their 12 corresponding squared terms) were regressed out [49,50]; (9) bandpass filtering: temporal filtering was applied to retain frequencies between 0.01–0.08 Hz, removing physiological noise outside this range.

### 2.4. Data analysis

#### 2.4.1. ALFF and FC analysis.

This study calculated ALFF and FC as indicators of resting-state neural mechanisms. Specifically, after smoothing but without filtering, ALFF values were computed for signals within the frequency band of 0.01–0.1 Hz across the three groups to minimize the influence of low-frequency drift and high-frequency physiological noise. To reduce interindividual differences in global ALFF levels, values were normalized by dividing by the global mean, yielding standardized zALFF maps. To further examine the functional connectivity patterns of regions showing significant differences in the ALFF analysis, seed-based resting-state functional connectivity analyses were performed. Specifically, peak coordinates of significant clusters identified in the ALFF analysis were used as seed regions, defined as spheres with a 6 mm radius, and the time series of voxels within each region of interest were extracted. Subsequently, DPABI was applied to compute voxel-wise Pearson correlation coefficients (r) between the seed time series and the whole brain at the individual level. To improve normality, correlation coefficients were transformed into Fisher’s z values.

For group-level analyses, head motion parameters, sex, and age were included as covariates, and ANCOVAs were performed on zALFF and zFC values across the three groups. Multiple comparisons were corrected using a two-tailed nonparametric permutation-based approach with 5,000 permutations combined with threshold-free cluster enhancement (TFCE) [51], with statistical significance defined at *p* < 0.05 with family-wise error (FWE) correction. This method rigorously controls the risk of false positives without relying on arbitrary voxel-wise or cluster-forming thresholds, thereby avoiding sensitivity differences introduced by threshold selection. Moreover, TFCE fully exploits the spatial continuity of brain signals, enabling robust identification of functional brain alterations [52,53].

Post hoc analyses of the mean zALFF and zFC values in regions identified by ANCOVAs were further conducted. Finally, brain regions that survived both the ANCOVA and post hoc tests were identified, and their mean zALFF and zFC values were examined in relation to NSSI behavioral and motivational measures using partial correlation analyses, with depressive symptom severity (SDS scores) controlled for, in order to reduce potential confounding effects of depression on the observed brain–behavior associations.

#### 2.4.2. Cross-validated prediction analysis.

We performed the 4-fold cross-validation analysis in R version 4.3.3 [54] using the *caret* and *pROC* packages. In the regression models, NSSI scores were defined as the dependent variable, while brain indices (i.e., ALFF and FC values from regions significantly associated with NSSI) served as independent variables. The dataset was randomly partitioned into four folds; three folds were used to train the linear regression model, and the remaining fold was used to validate it. This procedure was repeated four times, and the mean correlation coefficient *r*_(predicted, observed)_ between predicted and observed NSSI scores was calculated as the measure of predictive performance. To assess statistical significance, we adopted a nonparametric permutation test with 1,000 iterations under the null hypothesis that NSSI was unrelated to brain measures. The *p-*value was derived by calculating the proportion of permutation-derived *r* values greater than the observed *r*_(predicted, observed)_ and dividing this count by 1,000.

## 3. Results

### 3.1. Demographics and clinical measures

Demographic characteristics, scale scores, head motion parameters, and group comparison results are summarized in Table 1. No significant differences were observed among the three groups with respect to sex, age, or head motion parameters. ANOVA indicated significant group differences in SDS scores. Post hoc comparisons revealed no significant difference in SDS scores between the MDD with NSSI and MDD without NSSI groups (*Md* = 2.746, *SE* = 2.098, *p* = 0.393), whereas both MDD groups showed significantly higher SDS scores than the HC group (MDD with NSSI: *Md* = 31.940, *SE* = 2.050, *p* < 0.001; MDD without NSSI: *Md* = 29.194, *SE* = 2.087, *p* < 0.001).

### 3.2 ALFF results and behavior relevance

**Fig 1:**
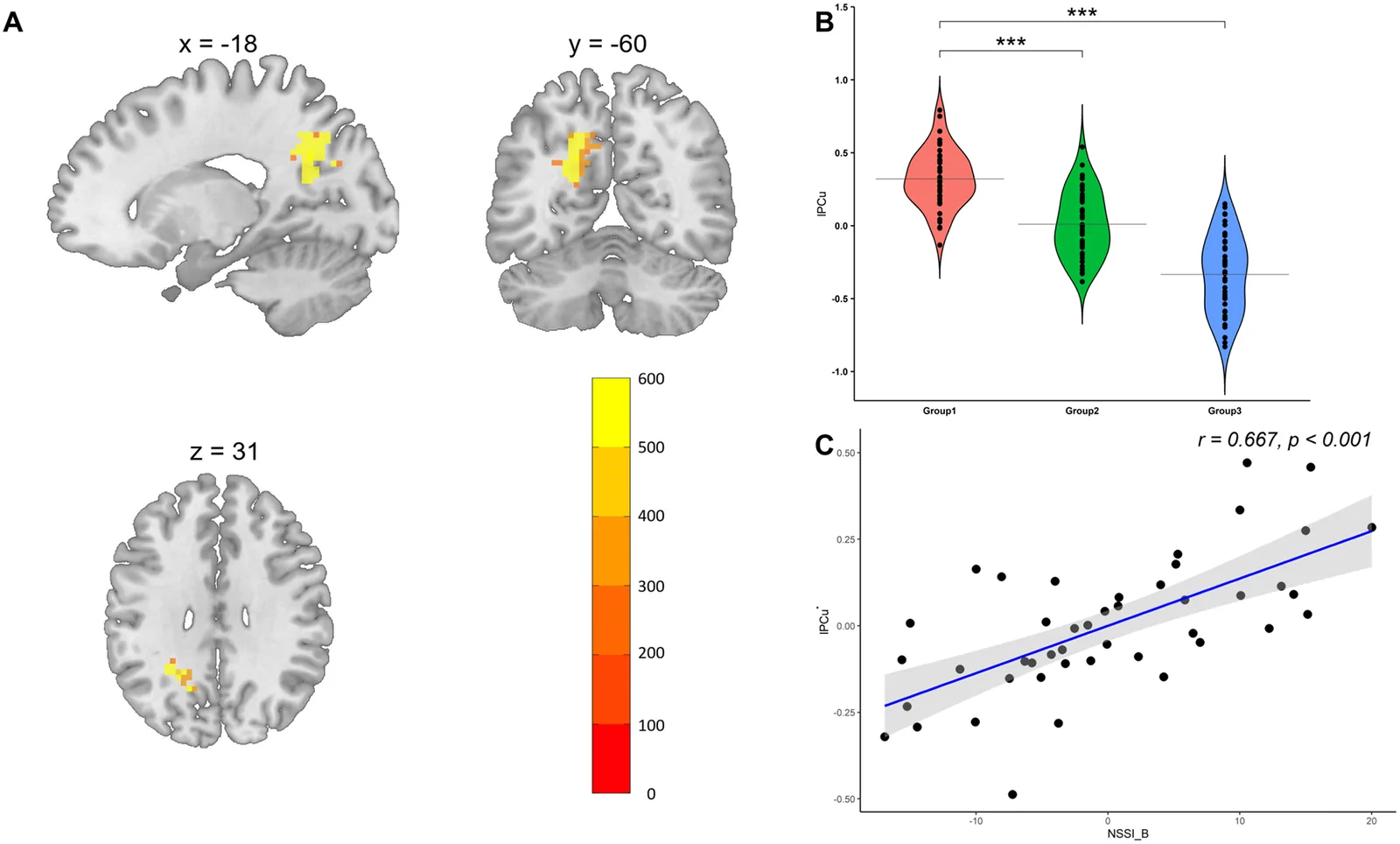
ALFF differences and brain–behavior association in the lPCu. (A) Significant ALFF differences in the lPCu identified using TFCE with FWE correction; the color bar indicates corrected F-values. (B) Group differences in ALFF values. (C) Partial correlation between lPCu ALFF and NSSI behavior in the MDD with NSSI group, controlling for SDS scores. Group1 = MDD with NSSI group; Group2 = MDD without NSSI group; Group3 = HC group. *Residuals after controlling for the covariate. ^***^ *p* < 0.001.

Results of the ALFF-based ANCOVA analyses and brain–behavior correlation analyses are presented in Table 2, Fig 1, and S1 and S2 Fig. Significant group differences in ALFF values were identified in three clusters, with peak coordinates located in the left rectal gyrus (lRectus), left precuneus (lPCu), and right inferior temporal gyrus (rITG). Post hoc comparisons revealed that ALFF values in all three regions differed significantly among groups. Table 2 summarizes the voxel extent, peak coordinates, ANCOVA *F* values, effect sizes, and post hoc comparison results for clusters showing significant group differences.

**Table 2:** Results of the ANCOVA and post hoc comparisons of brain functional measures.

Brain regions	Peak MNI (x, y, z)	Cluster size	*F*	η²_p_	Post hoc comparisons			
					G1 VS G2		G1 VS G3	
					*Md*	*SE*	*Md*	SE
**ALFF**								
lRectus	−3, 33, −25	129	100.447^***^	0.613	−0.280^***^	0.045	−0.625^***^	0.044
lPCu	−18, −60, 31	83	88.33^***^	0.582	0.314^***^	0.051	0.664^***^	0.051
rITG	63, −33, −15	7	47.418^***^	0.428	0.159^**^	0.041	0.393^***^	0.041
**FC**								
lPCu-rITG	43, −71, −4	106	48.289^***^	0.432	−0.216^***^	0.047	−0.460^***^	0.047
lPCu-lCuneus	−12, −86, 18	196	70.566^***^	0.526	0.285^***^	0.041	0.481^***^	0.041
lPCu-rPCu	9, −75, 54	34	46.794^***^	0.424	−0.266^***^	0.041	−0.385^***^	0.041

***Note***: lRectus = left rectal gyrus; lPCu = left precuneus; rITG = right inferior temporal gyrus; lCuneus = left cuneus; rPCu = right precuneus. G1 = MDD with NSSI; G2 = MDD without NSSI; G3 = healthy controls (HC). ^**^ *p* < 0.01, ^***^ *p* < 0.001. *Md* and *SE* represent the mean difference and standard error between groups in post hoc comparisons.

Partial correlation analyses further showed that, within the MDD with NSSI group, ALFF values in the lPCu were significantly associated with NSSI behavior (*r*_partial_ = 0.667, *p* < 0.001), but were not significantly associated with any of the three NSSI motivation (*r*_partial_ = −0.099, −0.081 and −0.161; *p* = 0.516, 0.595 and 0.290). In contrast, ALFF values in the left rectal gyrus and right inferior temporal gyrus did not show significant associations with NSSI behavior (*r*_partial_ = 0.021 and −0.042; *p* = 0.891 and 0.786) or motivation (*r*_partial_ ranged from −0.250 to 0.155; *p* ranged from 0.098 to 0.655). Results for the lPCu are shown in Fig 1, with results for the remaining regions and correlations with NSSI motivation provided in S1 and S2 Figs.

### 3.3 FC results and behavior relevance

**Fig 2:**
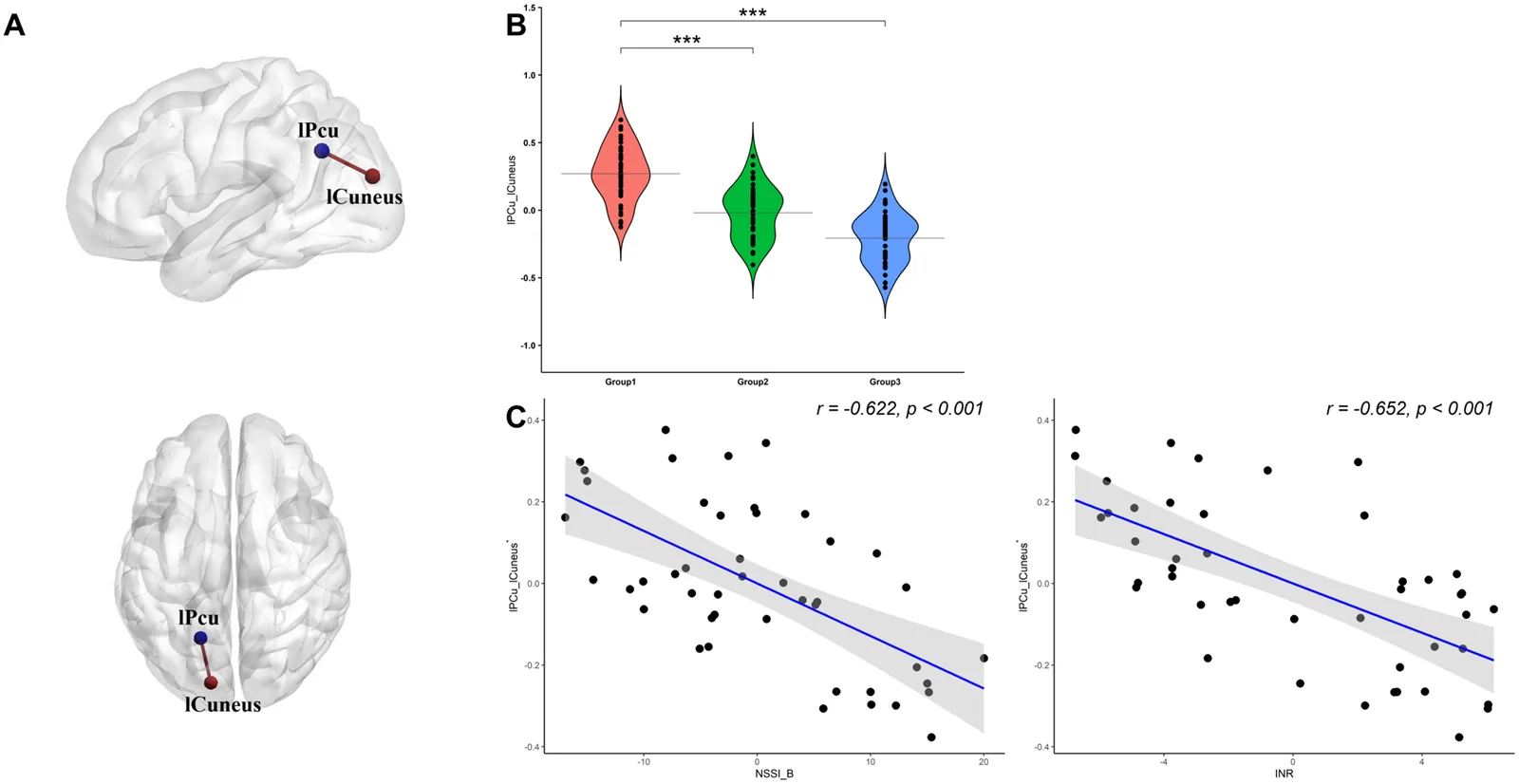
FC differences and brain–behavior association in the lPCu-lCuneus FC. (A) lPCu-lcuneus functional connectivity map (left lateral and superior dorsal views). (B) Group differences in FC. (C) Partial correlations with NSSI behavior and INR motivation (MDD with NSSI group, controlling for SDS).

Results of the FC-based ANCOVA and brain–behavior correlation analyses are presented in Table 2, Fig 2, and S3 and S4 Figs. Significant group differences in FC values were identified in three functional connections, all of which survived post hoc comparisons (see Table 2 and S3 Fig). However, only the left precuneus-left cuneus (lPCu–lCuneus) functional connectivity showed significant partial correlations with NSSI behavior (*r*_partial_ = −0.622, *p* < 0.001). In addition, significant partial correlations were also observed between lPCu–lCuneus FC and INR motivation (*r*_partial_ = −0.652, *p* < 0.001), as shown in Fig 2. In contrast, FC values in the remaining functional connections did not show significant associations with NSSI behavior (*r*_partial_ = −0.154 and 0.089; *p* = 0.312 and 0.561) or motivation (*r*_partial_ ranged from −0.243 to 0.076; *p* ranged from 0.108 to 0.999). Results for the lPCu-lCuneus FC are shown in Fig 2, with results for the remaining FCs and correlations with NSSI motivation provided in S3 and S4 Fig.

### 3.4. Cross-validated prediction

**Fig 3:**
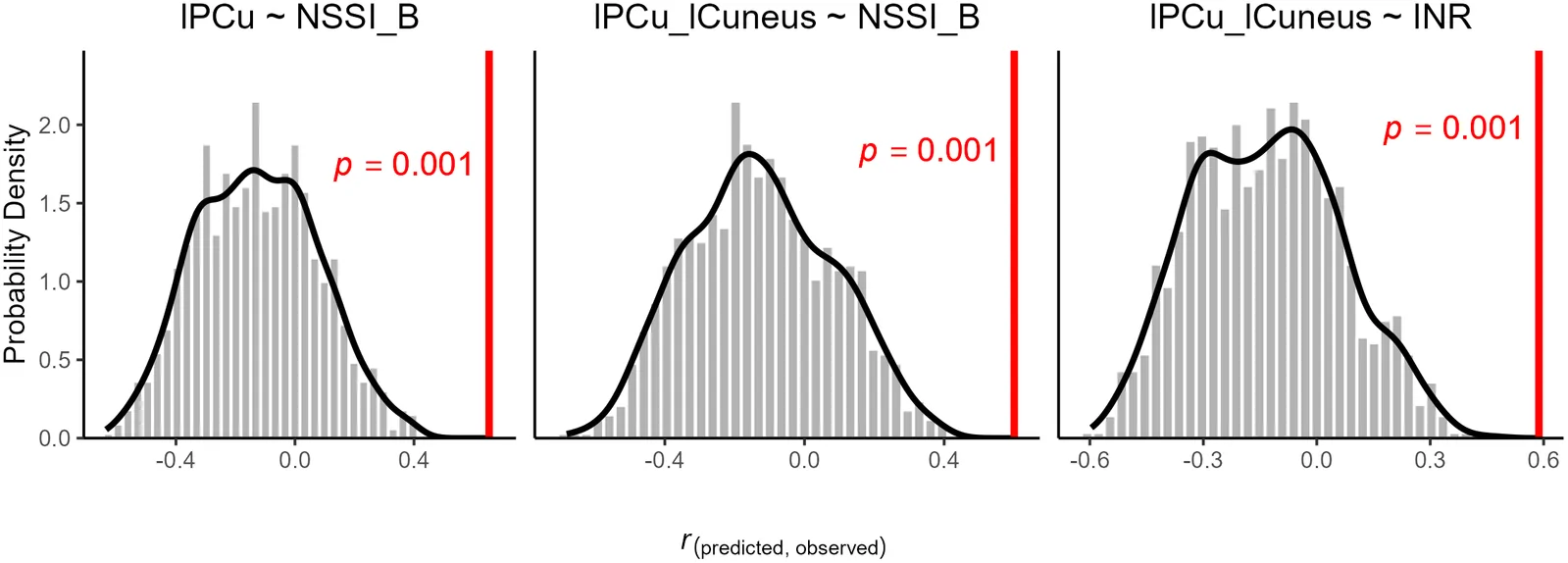
Cross-validated prediction of NSSI-related outcomes from lPCu ALFF and lPCu–lCuneus FC, controlling for SDS. Gray histogram: null distribution of the Pearson correlation (*r*) between predicted and observed values, generated from 1,000 random permutations of the outcome variable. Black density curve: kernel density estimate of the null distribution. Red vertical line: observed *r* from the true, unshuffled cross‑validated model. Red italic *p* value: proportion of permuted *r* values greater than or equal to the observed *r* (one‑tailed).

Moreover, prediction analysis demonstrated that ALFF values in the lPCu significantly predicted NSSI behavior (*r*_(predicted, observed)_ = 0.653, *p* = 0.001). Notably, prediction analyses demonstrated that lPCu–lcuneus FC significantly predicted both NSSI behavior (*r*_(predicted, observed)_ = 0.601, *p* = 0.001) and INR motivation (*r*_(predicted, observed)_ = 0.587, *p* = 0.001).These results are shown in Fig 3.

## 4. Discussion

In the current study, we used resting-state fMRI to examine the functional neural mechanisms of NSSI behaviors and motivations in adolescents with MDD, using ALFF and FC measures. To test the robustness of these brain–behavior associations, we further applied a machine learning-based cross-validation prediction approach. Results revealed that adolescents with MDD and NSSI exhibited significantly increased ALFF in the left precuneus compared with both MDD without NSSI and HC groups. Moreover, FC between the left precuneus and left cuneus was significantly higher in this group. Importantly, machine-learning cross-validation analyses demonstrated that these neural markers robustly predicted NSSI behaviors, and that the association between FC of the left precuneus–left cuneus and INR motivation for NSSI was stable and generalizable. These findings advance our understanding of the neural mechanisms underlying NSSI behaviors and motivations in adolescents with MDD, and provide neuroimaging evidence to inform risk identification and targeted interventions.

First, although early studies emphasized the role of the precuneus in visuospatial processing, recent neuroimaging research has increasingly highlighted its central involvement in a wide range of higher-order cognitive functions. As a key hub of the DMN [55], the left precuneus is broadly implicated in self-referential processing (e.g., rumination) [56], reward processing, emotion regulation, and mentalization—the capacity to understand one’s own and others’ mental states [57,58]. During adolescence, the development of the left precuneus and its functional connectivity is particularly pronounced. In this critical developmental period, both the intrinsic functions of the left precuneus and its connectivity with other networks undergo rapid maturation and reorganization [25,55]. These neural properties are essential for the development of self-concept, emotion regulation, and social adaptation during adolescence, and dysfunction within this region has been closely linked to depressive symptoms and suicide risk [57–59]. Thus, in adolescents with MDD and comorbid NSSI, resting-state activity of the left precuneus is markedly elevated, suggesting a functionally hyperactive state. This heightened activity may reflect excessive negative self-focus and ruminative thinking in contexts of emotional distress—for instance, continuous monitoring of one’s own emotional state, behavioral performance, and social feedback—which may amplify self-criticism, shame, and self-directed violence, thereby triggering or maintaining NSSI behaviors. Moreover, mentalization deficits associated with aberrant functioning of the left precuneus play a critical role in the onset and the maintenance of NSSI. Such impairments manifest not only in difficulties recognizing and regulating complex personal emotions, but also in misinterpretation of others’ intentions and emotional states. For example, individuals may perceive neutral or well-intentioned social feedback as rejection, criticism, or hostility, leading to interpersonal conflict, perceived social exclusion, and emotional distress. Abnormal activity in the left precuneus may reflect a dysregulation in the neural processes underlying mental state representation: although the brain attempts to enhance understanding of internal and others’ mental states, adolescents with MDD may lack effective mechanisms for regulatory and social-cognitive integration, resulting in emotional dysregulation, interpersonal misunderstanding, and affective instability, which may underlie the cognitive and neural processes contributing to the occurrence and persistence of NSSI behaviors.

Second, this finding—reflected in the significantly increased functional connectivity between the left precuneus and left cuneus in adolescents with MDD and comorbid NSSI—partially aligns with prior evidence implicating the cuneus in MDD and suicidal behaviors. Previous studies have reported structural and functional abnormalities of the cuneus in individuals with MDD [60–63], and altered volume or activity in this region has also been observed in bipolar disorder patients with suicidal ideation or a history of suicide attempts [62,63]. The cuneus, as a key component of the visual system, is involved in basic visual information processing and is associated with functions such as visual working memory and perceptual processing [61,64,65]. Importantly, the increased functional connectivity between the left precuneus and left cuneus was stably associated with both NSSI behavior and INR motivation. This finding suggests altered intrinsic coupling between two regions with distinct functional roles during rest. In adolescents with MDD, such atypical connectivity may reflect less efficient interaction between self-referential processing and visual information processing systems, which could be related to negative self-focused cognition and emotional dysregulation associated with NSSI. Clinically, individuals with MDD may be more likely to interpret mildly negative or neutral social cues as rejection or criticism, accompanied by repetitive rumination and self-blame, which in some cases may contribute to NSSI as a maladaptive coping strategy for emotional relief. Adolescence is a critical period of rapid development in social cognition and self-awareness, characterized by high neural plasticity [66]. In the context of MDD, pre-existing difficulties in emotion regulation and cognitive processing may interact with this ongoing developmental maturation, potentially contributing to altered functional connectivity between visual processing and self-referential regions, thereby exacerbating emotional dysregulation and NSSI behaviors.

Finally, this study provides neuroimaging evidence for the prediction and characterization of NSSI behaviors and their underlying motivations in adolescents with MDD. To begin with, heightened activity of the left precuneus and its increased functional connectivity with the left cuneus may serve as potential neurobiological markers associated with NSSI behaviors. These features may also be useful in future predictive and diagnostic models, thereby offering objective support for early identification and risk stratification. Moreover, the findings suggest potential neural systems that may serve as candidate targets for future intervention-related and mechanistic studies of NSSI. Previous research has demonstrated that repetitive transcranial magnetic stimulation (rTMS) can modulate functional connectivity within precuneus-related circuits in clinical populations [67,68]. In this context, the precuneus–cuneus circuitry identified in the present study may represent a candidate system for future investigations exploring whether neuromodulation can influence NSSI-related neural and behavioral processes. However, given the cross-sectional nature of the present study, these implications remain speculative and should not be interpreted as evidence for direct clinical efficacy or treatment recommendations.In future research, rTMS guided by rs-fMRI may provide a useful framework for investigating individualized modulation strategies at the single-subject level [69]. Within such a research framework, the left precuneus and its functional connectivity with the left cuneus could be examined as potential targets for experimental stimulation paradigms, with the aim of testing whether modulation of this circuit is associated with changes in neural activity and NSSI-related processes. Such approaches may include hypothesis-driven dual-site stimulation paradigms aimed at examining whether altering precuneus activity and its connectivity with the cuneus can influence the functional organization of self-referential and perceptual processing systems. These mechanistic studies may further help clarify whether such connectivity patterns are causally involved in cognitive and affective processes related to NSSI, including negative self-referential bias, self-critical processing, and self-injurious tendencies. Future longitudinal and interventional studies will be needed to determine the causal role of precuneus–cuneus connectivity in NSSI and to evaluate whether this circuit represents a viable target for neuromodulation-based mechanistic research.

Meanwhile, the present study has certain limitations, which may provide direction for future research. Future studies should expand the sample size to better validate the robustness and generalizability of the findings. Additionally, a more detailed examination of MDD subtypes could clarify whether the neural mechanisms underlying NSSI differ across them. Given the dynamic nature of neural development during adolescence, future research could adopt a stage-specific approach—examining early, middle, and late adolescence—to conduct stratified analyses and explore similarities and differences with the results of the present study spanning the entire adolescent period.

## 5. Conclusion

In summary, the present study elucidates the potential neural mechanisms underlying NSSI behaviors and motivations in adolescents with MDD. It highlights the critical functional role of the left precuneus in NSSI behavior and indicates that functional connectivity between the left precuneus and the left cuneus is centrally implicated in both NSSI behavior and INR motivation. Building on these findings, future research and clinical practice could advance early identification and prediction of NSSI risk and explore fMRI-guided, precision rTMS protocols, thereby providing neuroimaging evidence to inform strategies for reducing NSSI risk in adolescents with MDD.

## Supporting information

S1 Table Labels, short Name, full Content, and Chinese version of SDS items. (DOC)

S2 Table Labels, short Name, full Content, and Chinese version of ANSAQ items. (DOC)

S1 Fig ALFF differences (ANCOVA) and brain-NSSI behavior associations. (DOC)

S2 Fig ALFF-NSSI motivation associations in brain regions showing significant effects in ANCOVA. (DOC)

S3 Fig FC differences (ANCOVA) and brain-NSSI behavior associations. (DOC)

S4 Fig FC-NSSI motivation associations in brain regions showing significant effects in ANCOVA. (DOC)
