# Supplementary material for: Altered brain function associated with non-suicidal self-injury in adolescents with major depressive disorder: An rs-fMRI study
Source: PLoS One. 2026 Aug 20;21(8):e0356601. doi: 10.1371/journal.pone.0356601 (PMC13492744; doi:10.1371/journal.pone.0356601)
Supplement: Supplementary file 1 [file pone.0356601.s001.doc]

| **S1 Table.** Labels, short Name, full Content, and Chinese version of SDS items. | | |
| --- | --- | --- |
| **Label** | **Full context** | **Chinese version** |
| SDS1 | I feel down-hearted and blue | 我觉得闷闷不乐,情绪低沉 |
| SDS2 | Morning is when I feel the best | 我觉得一天中早晨最好 |
| SDS3 | I have crying spells or feel like it | 我经常哭泣或想哭 |
| SDS4 | I have trouble sleeping through the night | 我晚上睡眠不好 |
| SDS5 | I eat as much as I used to | 我吃得跟平常一样多 |
| SDS6 | I still enjoy close contact with the opposite sex as much as I did before | 我与异性密切接触时和以往一样感到愉快 |
| SDS7 | I notice that I am losing weight | 我发觉我的体重在下降 |
| SDS8 | I have trouble with constipation | 我有便秘的苦恼 |
| SDS9 | My heart beats faster than usual | 我心跳比平常快 |
| SDS10 | I get tired for no reason | 我无缘无故地感到疲乏 |
| SDS11 | My mind is as clear as it used to be | 我的头脑跟平常一样清楚 |
| SDS12 | I find it easy to do the things I used to | 我觉得经常做的事并没有困难 |
| SDS13 | I find myself restless and can’t keep still | 我觉得不安而平静不下来 |
| SDS14 | I feel hopeful about the future | 我对将来抱有希望 |
| SDS15 | I am more irritable than usual | 我比平常容易生气激动 |
| SDS16 | I find it easy to make decisions | 我觉得做出决定是容易的 |
| SDS17 | I feel that I am useful and needed | 我觉得自己是个有用的人,有人需要我 |
| SDS18 | My life is pretty full | 我的生活过得很有意思 |
| SDS19 | I feel that others would be better off if I were dead | 我认为如果我死了,别人会过得好些 |
| SDS20 | I still enjoy the things I used to do | 平常感兴趣的事我仍然感兴趣 |
