# Supplementary material for: Altered brain function associated with non-suicidal self-injury in adolescents with major depressive disorder: An rs-fMRI study
Source: PLoS One. 2026 Aug 20;21(8):e0356601. doi: 10.1371/journal.pone.0356601 (PMC13492744; doi:10.1371/journal.pone.0356601)
Supplement: Supplementary file 2 [file pone.0356601.s002.doc]

| **S2 Table.** Labels, short Name, full Content, and Chinese version of ANSAQ items. | | |  |
| --- | --- | --- | --- |
| **Label** | **Full context** | **Chinese version** |  |
| **Behavior subscale** | | | |
| NSSI1 | Intentionally pinching oneself | 故意掐伤自己 | |
| NSSI2 | Intentionally scratching oneself | 故意抓伤自己 | |
| NSSI3 | Intentionally banging one’s head against a hard object (e.g., wall, tree) | 故意用头撞较硬的物体( 如墙、树等) | |
| NSSI4 | Intentionally punching hard objects (e.g., wall, table, window, floor) | 故意用拳头打墙、桌子、窗户、地面等硬物 | |
| NSSI5 | Intentionally hitting oneself with a fist, slap, or hard object | 故意用拳头、巴掌或较硬的物体打伤自己 | |
| NSSI6 | Intentionally stabbing or pricking oneself (e.g., with needle, stapler pin, pen tip) | 故意扎或刺伤自己( 如用针、订书钉、笔尖等) | |
| NSSI7 | Intentionally cutting oneself (e.g., with razor blade, glass) | 故意割伤自己(如用刀片、玻璃等) | |
| NSSI8 | Intentionally biting oneself | 故意咬伤自己 | |
| NSSI9 | Intentionally pulling out one’s hair | 故意拽掉自己的头发 | |
| NSSI10 | Intentionally burning or scalding oneself (e.g., with cigarette, boiling water, lighter, match) | 故意烧伤或烫伤自己(如用烟头、开水、打火机或火柴等) | |
| NSSI11 | Intentionally rubbing skin with an object until bleeding or bruising | 用东西故意摩擦皮肤使其出血或淤血 | |
| NSSI12 | Intentionally carving words or symbols into the skin (excluding tattoos) | 故意在皮肤上刻字或符号(不包括纹身) | |
| **Function subscale** | | |  |
| NSSI13 | Expressing one’s anger | 表达自己的愤怒 |  |
| NSSI14 | Escaping from unpleasant or upsetting situations (e.g., avoiding school, homework, or labor) | 为了逃避自己不喜欢，或让自己不开心的事情(如逃避上学、做作业或劳动等) |  |
| NSSI15 | Making oneself feel less lonely | 让自己觉得不孤独 |  |
| NSSI16 | Relieving stress or anxiety | 缓解压力或焦虑的心情 |  |
| NSSI17 | A way of self-punishment or atonement | 自我惩罚或赎罪的方式 |  |
| NSSI18 | Bringing pleasure or enjoyment, making oneself feel good | 能带来快乐、享受，让自己感觉很好 |  |
| NSSI19 | Gaining control over oneself and calming down | 控制自己，使自己冷静下来 |  |
| NSSI20 | Attracting others’ attention | 吸引别人的注意 |  |
| NSSI21 | Taking revenge on others | 报复别人 |  |
| NSSI22 | Preventing oneself from hurting others | 只有这样自己才不会去伤害别人 |  |
| NSSI23 | Protecting oneself from others’ attacks | 保护自己不受别人的攻击 |  |
| NSSI24 | Helping oneself stop unwanted thoughts or ideas | 能帮助自己停止不好的想法或念头 |  |
| NSSI25 | Because friends have done it before | 朋友曾经这样做过 |  |
| NSSI26 | Having the urge to hurt oneself and being unable to stop | 有伤害自己的欲望，且无法停止 |  |
| NSSI27 | Gaining understanding from others | 获得他人的理解 |  |
| NSSI28 | Coping with feelings of sadness or disappointment | 应对悲伤、失望的情绪 |  |
| NSSI29 | Expressing despair and helplessness | 表现自己的绝望和无助 |  |
| NSSI30 | Forcing others to make changes | 让其他人做出改变 |  |
| NSSI31 | Escaping from numbness or unreality | 从麻木和虚幻中逃脱出来 |  |
