# Supplementary material for: Altered brain function associated with non-suicidal self-injury in adolescents with major depressive disorder: An rs-fMRI study
Source: PLoS One. 2026 Aug 20;21(8):e0356601. doi: 10.1371/journal.pone.0356601 (PMC13492744; doi:10.1371/journal.pone.0356601)
Supplement: Supplementary file 3 [file pone.0356601.s003.doc]

**S1 Fig. ALFF differences (ANCOVA) and brain-NSSI behavior associations.** (a) Significant ALFF differences identified using TFCE with FWE correction; the color bar indicates corrected F-values. (b) Group differences in ALFF values. (c) Partial correlation between brain and NSSI behavior in the MDD with NSSI group, controlling for SDS scores. Group1 = MDD with NSSI group; Group2 = MDD without NSSI group; Group3 = HC group. lRectus = left rectal gyrus; lPCu = left precuneus; rITG = right inferior temporal gyrus. NSSI-B = Adolescent Non-suicidal Self-injury Assessment Questionnaire behavior subscale score. ^***^ *p* <0.001.
