# Supplementary material for: Altered brain function associated with non-suicidal self-injury in adolescents with major depressive disorder: An rs-fMRI study
Source: PLoS One. 2026 Aug 20;21(8):e0356601. doi: 10.1371/journal.pone.0356601 (PMC13492744; doi:10.1371/journal.pone.0356601)
Supplement: Supplementary file 4 [file pone.0356601.s004.doc]

**S2 Fig. ALFF-NSSI motivation associations in brain regions showing significant effects in ANCOVA.** SPR = social-positive reinforcement; INR = intrapersonal-negative reinforcement; EE = emotion expression.
