# Supplementary material for: Altered brain function associated with non-suicidal self-injury in adolescents with major depressive disorder: An rs-fMRI study
Source: PLoS One. 2026 Aug 20;21(8):e0356601. doi: 10.1371/journal.pone.0356601 (PMC13492744; doi:10.1371/journal.pone.0356601)
Supplement: Supplementary file 5 [file pone.0356601.s005.doc]

**S3 Fig. FC differences (ANCOVA) and brain-NSSI behavior associations.** (a) Significant FC differences identified using TFCE with FWE correction; the color bar indicates corrected F-values. (b) Group differences in ALFF values. (c) Partial correlation between brain and NSSI behavior in the MDD with NSSI group. ^*^Residuals after controlling for the covariate. lCuneus = left cuneus; rPCu = right precuneus.
